# Crimean-Congo Hemorrhagic Fever Virus: An Emerging Threat in Europe with a Focus on Epidemiology in Spain

**DOI:** 10.3390/pathogens13090770

**Published:** 2024-09-06

**Authors:** María Eslava, Silvia Carlos, Gabriel Reina

**Affiliations:** 1 Microbiology Department, Clínica Universidad de Navarra, 31008 Pamplona, Spain; meslavalaho@alumni.unav.es (M.E.); gabi@unav.es (G.R.); 2 Department of Preventive Medicine and Public Health, Universidad de Navarra, 31008 Pamplona, Spain; 3 IdiSNA, Navarra Institute for Health Research, 31008 Pamplona, Spain

**Keywords:** Crimean-Congo hemorrhagic fever virus, Europe, emerging infectious diseases, tick borne disease, *Hyalomma*

## Abstract

Crimean-Congo hemorrhagic fever (CCHF) is a tick-borne disease transmitted by ticks of the genus *Hyalomma* and caused by a virus of the *Nairoviridae* family. In humans, the virus can generate different clinical presentations that can range from asymptomatic to mild illness or produce an hemorrhagic fever with a mortality rate of approximately 30%. The virus pathogenicity and the lack of effective treatment or vaccine for its prevention make it an agent of concern from a public health point of view. The main transmission route is tick bites, so people most exposed to this risk are more likely to become infected. Another risk group are veterinarians and livestock farmers who are in contact with the blood and other fluids of animals that are mostly asymptomatic. Finally, due to its first phase with a non-characteristic symptomatology, there exists a risk of nosocomial infection. It is endemic in Africa, the Balkans, the Middle East, and those Asian countries south of the 50th parallel north, the geographical limit of the main vector. Recently, autochthonous cases have been observed in areas of Europe where the virus was not previously present. Human cases have been detected in Greece, Bulgaria, and Spain. Spain is one of the most affected countries, with a total of 17 autochthonous cases detected since 2013. In other countries, such as France, the virus is present in ticks and animals but has not spread to humans. A high-quality epidemiological surveillance system in these countries is essential to avoid the expansion of this virus to new areas and to limit the impact of current cases.

## 1. Introduction

Crimean-Congo hemorrhagic fever (CCHF) is a disease caused by *Orthonairovirus haemorrhagiae*, a virus from the *Nairoviridae* family, *Bunyaviridae* order. It is primarily transmitted by ticks, generally of the genus *Hyalomma*. In humans, the virus can cause a clinical presentation ranging from asymptomatic to mild illness or can result in hemorrhagic fever with a mortality rate of approximately 30% [1].

Due to its high lethality, lack of treatment, high transmission risk, and the virus’s ability to mutate, it is a cause of significant concern.

The disease is endemic in Africa, the Balkans, the Middle East, and Asian countries situated south of the 50th parallel north, which marks the geographical limit of the primary vector [2].

The virus can be transmitted through tick bites or direct contact with the blood, excretions, and secretions of infected patients and animals that act as virus hosts [3].

Those at highest risk of contracting the disease include healthcare workers, due to the risk of nosocomial infection, and individuals who are more exposed to tick bites due to their occupations, such as farmers and livestock handlers. People working in slaughterhouses or those who may be exposed to animal blood or other fluids, such as veterinarians, are also at increased risk [1].

In recent years, CCHFV (Crimean Congo Hemorrhagic Fever Virus) has been detected in regions of Europe outside the Balkans. This is due to global warming, as migratory birds transport ticks to non-endemic areas, and international trade, which can represent a public health risk [4].

In this context, the objective of this review is to understand the current situation of the virus in Europe, focusing on its epidemiology and geographical distribution, as well as to gain a deeper understanding of the virus’s variability, the characteristics of the disease, form of infection, and transmission. Additionally, this review aims to explore the different diagnostic methods and existing treatments to better comprehend this emerging disease and the impact of its arrival in Europe.

## 2. Materials and Methods

### 2.1. Search Strategy

For this literature review, articles were searched from the year 2000 to 2024. The search began on 5 February 2024 and ended on 5 May 2024. Out of 441 initial articles, through various selection criteria explained in Section 2.4, the number was reduced to 40 articles for a full review to complete the work.

It is important to note that some of the articles not selected were saved for potential use once the work began. 

**Figure 1:**
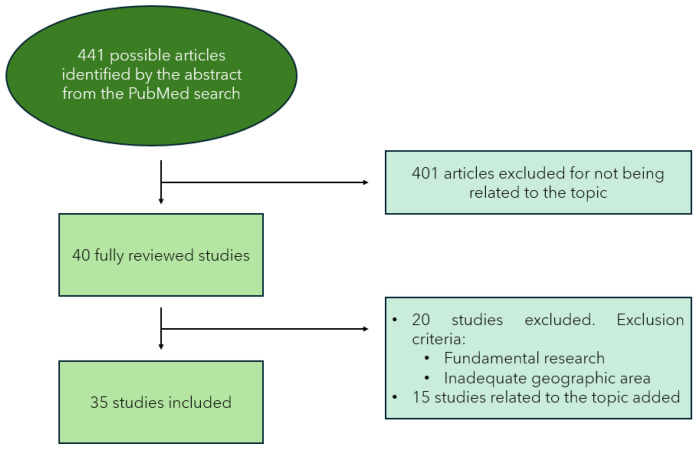
Schematic summary of the search strategy conducted for the literature review. The included studies date from 2000 to 2024.

During the reading of the articles and writing of the work, some of the initially selected articles were discarded, and new ones related to the topics discussed in the paper were added (Figure 1).

### 2.2. Documentary Sources

To obtain the necessary information for the work, the main databases used were PubMed and Web of Science.

### 2.3. Used Descriptors

The following descriptors were introduced to reach the selected articles: “Crimean Congo” resulting in 2061 articles; “Crimean Congo and Europe” with 441 articles, from which the search was initiated; “Crimean-Congo hemorrhagic fever and emerging disease in Europe” with 117 articles. Many of these had been previously selected, but it allowed the addition of six more articles to review.

### 2.4. Selection Criteria

The selection criteria, in addition to the years and search criteria previously mentioned, were based on the titles and keywords of the articles, followed by reading the abstracts. Articles focused on describing the epidemiology of the virus in endemic areas or outside the selected geographic area were discarded. Primarily, original articles were sought, and although reviews were avoided, some were used because their characteristics could provide a general understanding of the disease.

## 3. Characteristics of the Crimean Congo Hemorrhagic Fever Virus

### 3.1. Structure

CCHFV is characterized by a single-stranded, negative-sense RNA genome enclosed within an envelope. Its genome is divided into three distinct segments: small (S), medium (M), and large (L). These segments encode four structural proteins: the viral nucleocapsid protein (N), glycoproteins (Gn and Gc), and the polymerase protein (RNA-dependent RNA polymerase), respectively. The M segment specifically encodes both structural glycoproteins, Gn and Gc, along with nonstructural proteins, including NSm (non-structural M protein) and MLD (mucin-like domain) [1,5].

**Figure 2:**
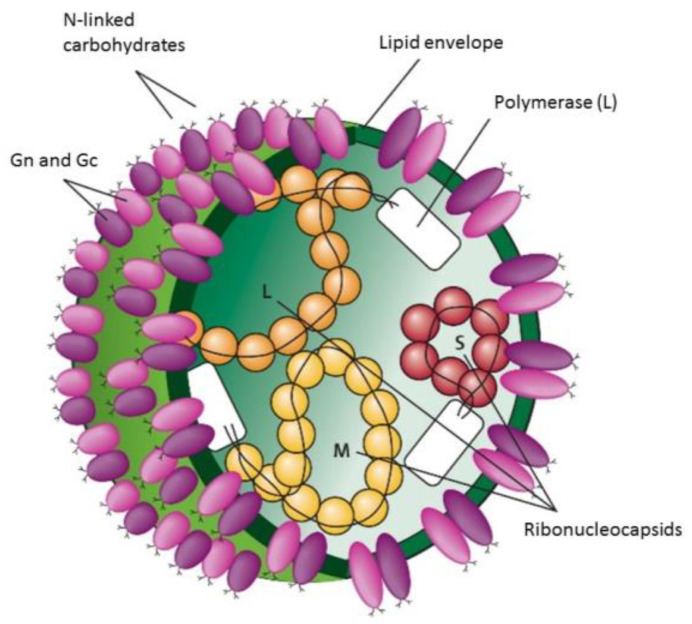
Schematic presentation of the CCHFV structure [1].

These segments are encapsulated by a nucleoprotein (NP). The structure of the virus is observed in Figure 2.

The glycoproteins Gn and Gc result from the proteolytic processing of a protein encoded by the M segment, an essential process for the virus to reach maturity [6]. These glycoproteins are responsible for recognizing receptors on cells susceptible to infection by this virus. Once the virus binds to these receptor sites, it is internalized by endocytosis, and the virus replicates within the cytoplasm of the cell. Mature virions are then released from the endoplasmic reticulum into cytoplasmic vesicles located in the Golgi apparatus [1,5].

Furthermore, its structure includes an enzyme necessary for initiating transcription and genome replication within the host cell, the RNA-dependent RNA polymerase, encoded by the L segment.

Its three-segmented genome provides the virus significant genetic variability and recombination potential.

### 3.2. Genetics

There are different viral strains, and within the previously mentioned segments (S, M, and L), there is a genetic variation of 20%, 31%, and 22%, respectively. This characteristic makes the CCHFV one of the arthropod-borne viruses with the highest genetic diversity [5].

**Figure 3:**
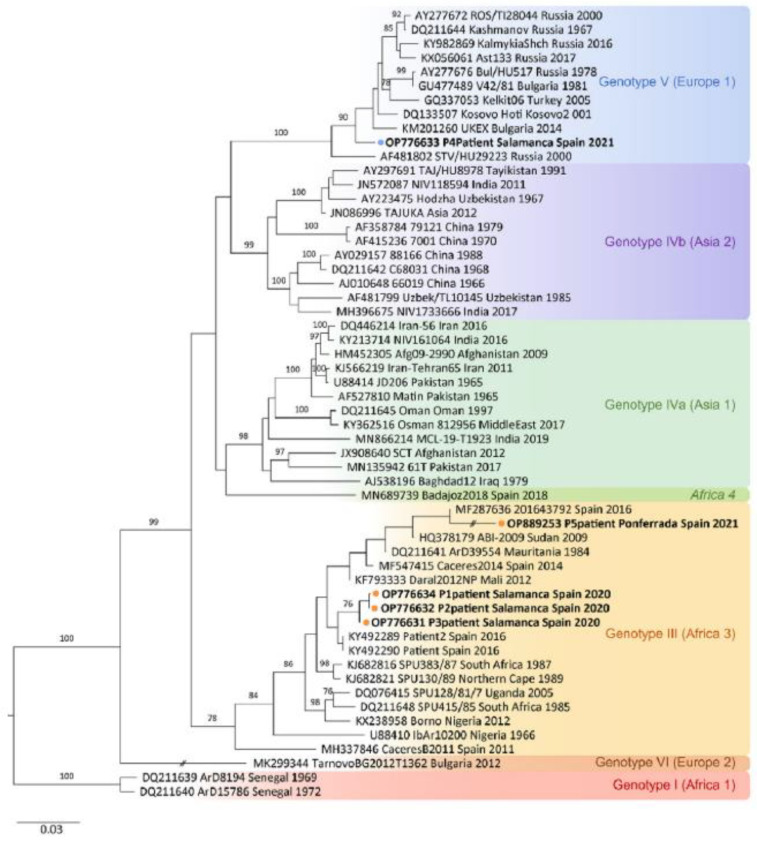
Phylogenetic tree of the CCHFV [5].

In this context, it is notable that phylogenetic analysis of both complete and partial sequences of the S segment distinguishes seven groups of CCHFV (Figure 3).

The genotypes are named based on the geographical area where the virus was initially isolated. These are: Africa 1 (genotype I), Africa 2 (genotype II), and Africa 3 (genotype III); Asia 1 (genotype IVa) and Asia 2 (genotype IVb); Europe 1 (genotype V) and Europe 2 (genotype VI) [5,7].

Each genetic lineage is associated with a geographic area. However, strains have migrated between regions due to migratory birds, unregulated wildlife trade, livestock import and export, and the widespread global movement of humans today [7].

Focusing more on autochthonous cases in Europe, genotype III of CCHFV was detected in ticks collected from deer in Spain in 2010. A phylogenetic analysis of the virus obtained from the serum of patients infected with CCHFV in 2016 indicated that the responsible strain was Africa 3 (genotype III). It is hypothesized that it reached Spain via migratory birds from Morocco, showing that this strain is circulating in Southwestern Europe [7]. A more recent study of a case detected in Salamanca (Spain) in 2018 shows that the strain causing the virus was genotype V, which was likely introduced by the movement of livestock from Central and Eastern Europe, where the disease is endemic [8].

## 4. Epidemiology

CCHF is primarily transmitted to humans through a tick bite, usually from the genus *Hyalomma*, although it has also been found in other tick genus such as *Rhipicephalus*, *Ornithodoros*, *Boophilus*, *Dermacentor*, and *Ixodes*.

Infection can also result from direct contact with the blood or tissues of a host infected with this virus (either human-to-human or animal-to-human transmission) [9]. However, the route of transmission to humans is not yet clearly established.

The geographical distribution of CCHFV coincides with the distribution of its main transmitting vector, the *Hyalomma* tick, which is limited to 50° North latitude [10]. The primary vector in Europe is *H. marginatum* [4], although CCHFV was found in *H. lusitanicum* in Spain in 2010 [3], another of the main vectors of the virus. A recent model has found an increased risk for CCHF occurrence in warmer and drier areas, which are the preferred habitats of the vector, with higher abundance during the summer months. Stable populations of these ticks are found in Southern Europe, primarily in the warm regions of the Mediterranean [11].

One characteristic of this type of tick is that they require two hosts to complete their life cycle. In the case of migratory birds, the ticks are transported in their immature stages on the bird, feeding on it and potentially transmitting CCHFV. Once the birds reach Europe, the ticks, now mature, attach to a second host, either an animal or a human. Therefore, migratory birds seem to be one of the main pathways for the virus to enter Europe [10,12].

The disease only develops in humans, but the virus circulates in various domestic and wild animals, notably cattle (79.1%), sheep (75%), goats (66%), and horses (58.8%) [10,13]. Considering this, it is important to note that adult tick transmission through livestock is believed to be another primary route for introducing CCHFV into previously unaffected areas [14].

People at higher risk of infection are farmers and shepherds, due to their greater exposure to ticks; livestock handlers, because of their contact with main animals that can act as asymptomatic hosts of this disease; and healthcare workers, due to the high risk of nosocomial infections [15].

**Figure 4:**
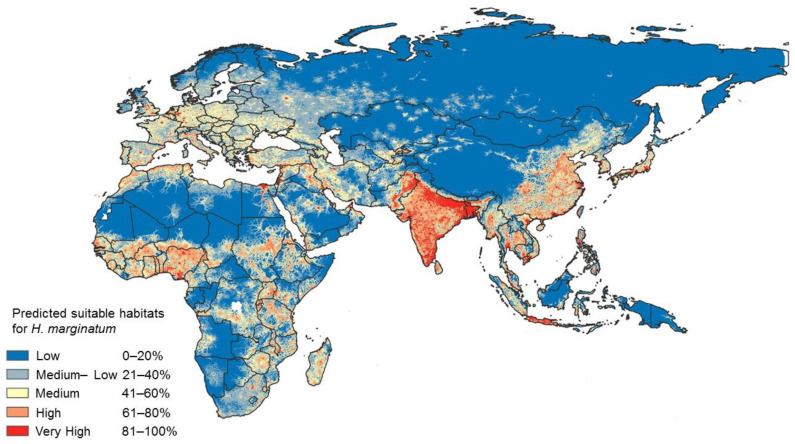
Predicted potential geographic distribution of *Hyalomma marginatum* on a global scale [17].

Historically, *H. marginatum* has been found across the Mediterranean, Balkan countries, Ukraine, and southern Russia. Recent ecological niche modelling suggests a broader potential distribution across Europe, extending into Central Europe and even southern Scandinavian regions. Globally, Hyalomma ticks have a wide range, spanning from western China and South Asia to the Middle East, Southeast Europe, and Africa. The predicted global habitats suitable for *H. marginatum* are depicted in Figure 4, highlighting the potential for the further spread of CCHF, especially in regions with favourable climatic conditions [16].

An ECDC report indicates that some countries without reported CCHF cases still possess the environmental conditions for autochthonous disease transmission. The highest risk areas are in Mediterranean countries, such as France, Italy, and the southern Balkans. Rising temperatures due to climate change could drive the expansion of both the vector and the disease into northern Europe [17,18].

The Epidemic Intelligence from Open Sources (EIOS) initiative has demonstrated its effectiveness in extracting information on outbreak locations, which can be used to identify high-risk areas. These online data on disease risk can assist decision-makers, public health authorities, and veterinary services in prioritizing areas where surveillance is most needed [11].

A more detailed list of the epidemiology in various European countries is provided below, with a deeper description of those with reported human infection cases.

a. Bulgaria

The disease appears to be endemic, and most cases in Europe in recent years have been reported from this country [9].

A total of 37 cases have been reported since the epidemiological surveillance records began in 2013, with 7 resulting in patient deaths [16]. However, the first cases of the disease in the country date back to before these records.

To contextualize the importance of risk factors, we will discuss four cases of CCHF that occurred in Bulgaria in 2008. The first two cases were men, aged 40 and 30, who had removed ticks from a cow without wearing gloves or taking other precautions. The third case was a nurse who cared for them, and the fourth was a close relative of one of the infected individuals who had been in contact with his blood [19].

b. Hungary

Only one case of CCHFV infection in humans was reported in 2004, although the virus was detected in 1973 through a seroprevalence study in both sheep and cattle [20].

In a 2012 study where 2163 adult ticks were collected from cattle and wild animals, two male *H. marginatum* rufipes ticks, which act as vectors for this virus, were found. No tests were conducted to determine if the ticks carried the virus. It is worth noting that the area where the ticks were found is near where the person who contracted the infection in 2004 came from [20].

c. Greece

Two human cases have been described in this country, and it appears that one of them was imported. 

The first case was reported in 2008 in a 46-year-old woman engaged in agricultural activities [15]. Recently, a single case in 2018 was reported and seems to be related to a travel to Bulgaria [9].

d. United Kingdom

No autochthonous cases have been detected. However, in 2014, an imported human case from Bulgaria was detected [16].

According to a 2016 study, there is minimal, though still significant, risk of the virus entering through migratory birds carrying *H. marginatum* ticks, as only 1% of birds entering through the migratory bird route from Africa and Spain carried *H. marginatum.* This underscores the importance of good monitoring to control the situation in the future [21].

e. Sweden

No autochthonous or imported human cases have been detected since records began.

A study conducted from July 2018 to January 2019 involved people reporting to health authorities if they found ticks on animals or people. A total of 41 adult Hyalomma ticks were found for the first time, but they did not carry CCHFV. An important factor mentioned in the study is that the summer of 2018 was the hottest on record at that time [22].

f. France

No autochthonous human infection cases have been described in this country. In 2004, there was an imported case from Senegal [23].

A study was conducted to determine the infection rate in ticks. Samples were taken from farm animals, specifically cattle and horses, in the eastern Pyrenees area in May 2022 and April 2023. A total of 1998 ticks were collected, and the proportion of infected ticks ranged from 3.1% to 55.8% (median: 7.4%), with most positive cases found in ticks on cattle [24].

In Corsica, two serological studies on farm animals were conducted during the periods 2014–2016 and 2019–2020. A total of 3890 and 6070 serum samples from cattle, sheep, and goats were analysed, respectively. A 13–16% positive prevalence was detected in cattle compared to a 2–3% positivity in sheep and goats [23].

A serological study was also conducted on wild animals (wild boars, foxes, deer, roe deer, and mouflons), with serum samples collected from various regions: Hérault, Upper Corsica, Lozère, and the Pyrenees. Samples could be from the period 2008–2022. Out of 2383 animals, 46 (1.9%) had antibodies against CCHFV [23].

g. Germany

No human cases of the disease have been described.

In 2007, a *Hyalomma marginatum* tick (the main vector of CCHFV) was found on a patient’s leg, but it did not carry CCHF [25].

h. Italy

No human cases of the disease have been reported.

Nevertheless, due to its Mediterranean climate and being a route for various migratory birds, there is a high risk of the virus entering the country.

From March to May 2017, a study was conducted on the Italian island of Ventotene to examine ticks on birds and determine if they were infected with CCHFV. A total of 5095 birds were examined, and ticks in different stages (adults, nymphs, and larvae) were collected. RNA of CCHFV was detected in a nymph of the genus *H. rufipes*. The same study revealed that the strain of the virus was genotype III [12].

In 2018, a serological study on sheep was conducted to determine the presence of antibodies against CCHF. The study was carried out in coastal areas of central Italy (Grosseto, Latina, Rome, and Viterbo) and the results were negative. This indicated that, at that time, either the virus had not been introduced to the study region or there had been no autochthonous transmissions to vertebrate hosts [14].

The following table summarizes the positive human cases reported in Europe in recent years (Table 1).

### Epidemiology in Spain

A total of 17 human cases of CCHF have been diagnosed in Spain, 14 of which are listed in Table 1, and three more occurred before 2017.

**Figure 5:**
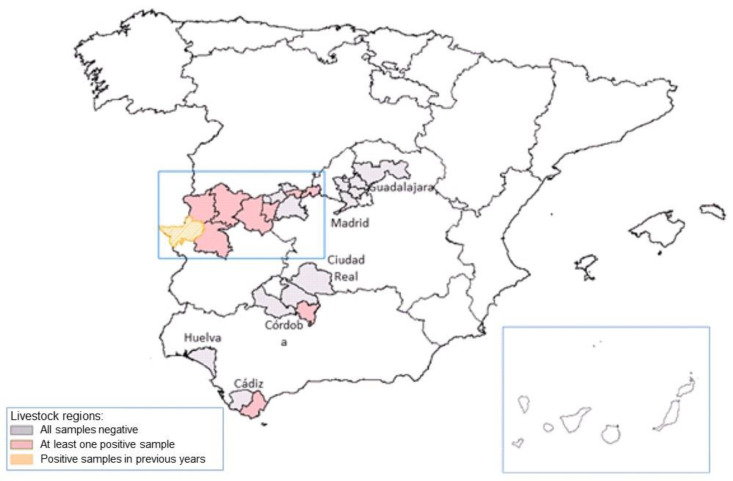
Studies of the Crimean-Congo haemorrhagic fever virus in ticks collected from animals (period: September 2016–February 2017) and vegetation (period: May 2017–October 2017) [27].

In 2010, *H. lusitanicum* ticks were detected in the province of Cáceres, which tested positive for CCHFV [26], representing the first finding of this virus in the Iberian Peninsula. As shown in Figure 5, the presence of the virus has been confirmed in ticks in the regions of Extremadura, Castilla-La Mancha, Castilla y León, and Andalusia. Additionally, in 2018, these autonomous communities detected seropositive rates of 70% in wild animals (wild ruminants, wild boars, hares, and rabbits) and 16% in domestic animals (extensive livestock: cattle, sheep, and goats) [27].

Of the 17 confirmed cases in Spain, 14 occurred between 2017 and 2024. Two of the remaining three cases occurred in 2016, and an additional case from 2013 was discovered through a retrospective study [28]. Six of the cases resulted in the patient’s death (35.3% mortality rate in Spain).

In 2016, the first two autochthonous cases of the disease were diagnosed in Spain. One man contracted the disease from a tick bite in the province of Ávila. The next case, also in 2016, was a nosocomial infection of the nurse who cared for the first patient and had direct contact with his blood [3].

Since the first two cases reported in 2016, fifteen additional cases have been documented to date, as shown in Table 2. In 2024 alone, four cases have been reported. One of these involved an elderly man who contracted the disease from a tick bite in Salamanca and passed away on 1 May 2024 [29]. The most recent three cases, reported in the summer of 2024, involved men aged 46 to 74 who contracted the disease from tick bites in Toledo, Seville, and Caceres, respectively, during July and August 2024. All three survived [30].

With the exception of a few cases reported in Extremadura, the majority of infections occurred in the autonomous community of Castilla y León, where there were seven cases in Salamanca, three in León, and two in Ávila. This region is considered high-risk due to the significant presence of *Hyalomma lusitanicum* vectors infected with CCHFV [27,28,31].

A retrospective study conducted in 2021 identified an additional CCHF case in Spain from 2013, before the first reported case. A woman had a disease following a tick bite in Southern Castilla y León. At that time, there was no record of CCHF cases in the country, so it was not investigated. In 2021, a serum sample tested positive for antibodies against the CCHFV in both an ELISA test and an immunofluorescence assay. Additionally, blood and serum samples taken in 2013 when the patient visited the hospital were found to contain CCHFV genome, confirming the first human case of the infection in Spain [28].

**Figure 6:**
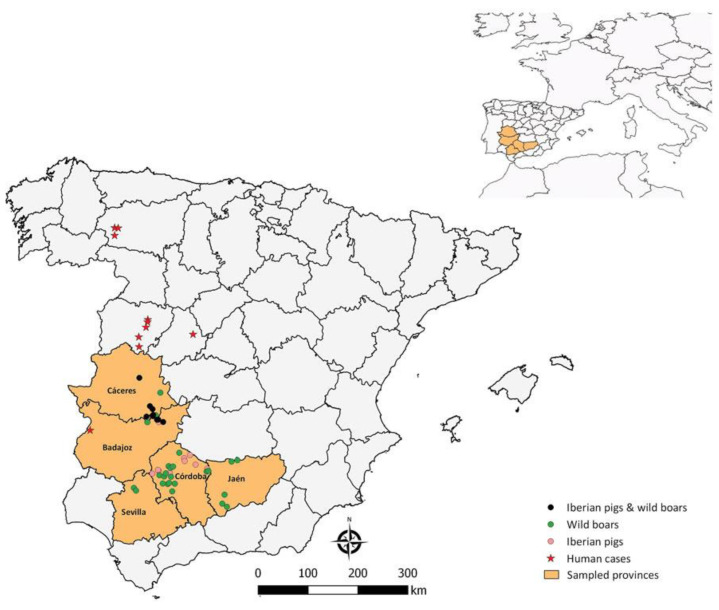
Seroprevalence in suids in the Iberian Peninsula in the provinces of Cáceres, Badajoz, Sevilla, Córdoba, and Jaén. Distribution of reported human cases in Spain [32].

In May 2024, a study was conducted on suids (wild boars and pigs) in five provinces in Southwestern Spain (Figure 6). Samples were collected in different periods: 2015–2016 and 2020–2021 for wild boars and 2017–2019 for pigs [32].

Of the 518 suids studied, 113 showed antibodies against the studied agent, demonstrating a seroprevalence of 21.8%. Seroprevalence was significantly higher in wild boars, 39.7%, compared to 2.8% in pigs. Córdoba was the province with the highest virus seroprevalence compared to Badajoz, an area where human cases have been detected (Figure 6).

Seropositivity was found in all years and in all provinces where samples had been collected, indicating an endemic distribution of the infection in southwestern Spain [32].

## 5. Pathogenicity

Regarding the symptoms and clinical signs of the disease, it should be noted that humans are the only hosts in which the disease manifests. Talking about animals, the infection is, in most of the cases, asymptomatic. The viremia in vertebrate animals necessary to allow tick infection and therefore maintain the virus in nature lasts around one week [23]. The short period of viremia in animals implies that the mechanism of virus transmission to humans through these animals has less relevance [27].

CCHFV infection generally has four phases: incubation, pre-haemorrhagic phase, haemorrhagic phase, and convalescent period [1]. 

**Figure 7:**
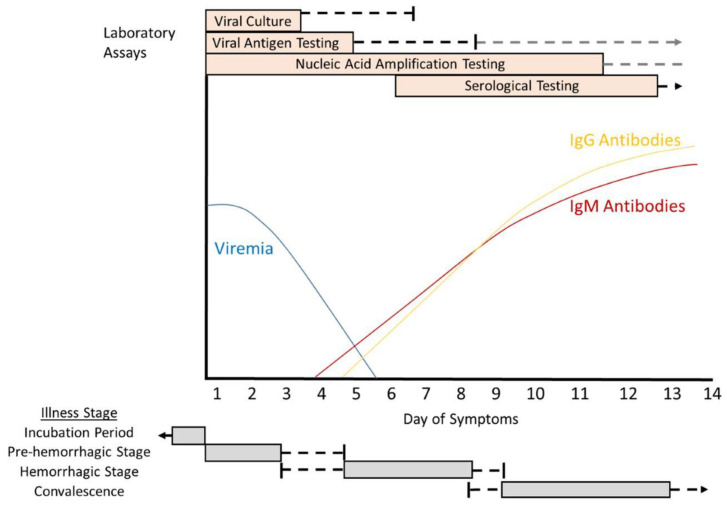
Diagnostic tests in non-fatal acute human infection due to CCHFV. The grey boxes refer to the duration in days of each stage of the disease. Patients with fatal CCHF infections may have prolonged viremia and may not develop antibodies against CCHFV. The period of greatest utility for the tests is shown in the orange box [34].

The incubation period, from the exposure to the virus until the disease manifests, usually lasts between 3 and 7 days. The onset of the disease is usually sudden and is characterized by intermittent fever with shivers and shakes, headache, dizziness, irritated eyes, myalgia, sore throat, and in some cases, abdominal pain, diarrhoea, and vomiting. In many cases, patients exhibit injected conjunctivae and redness, which are characteristic symptoms of the pre-haemorrhagic period, lasting between 1 and 7 days. However, symptoms vary depending on the case and the patient [33]. Figure 7 schematically shows the duration of each of these phases.

The haemorrhagic period lasts between 3 and 5 days. This phase is marked by petechial rash on the trunk and limbs, as well as the appearance of bruises. The most common bleeding episodes affect the gastrointestinal tract, uterus, urinary tract, and respiratory tract [33]. Finally, the convalescent period lasts about 10 to 20 days after the disease has resolved.

The pathogenicity of the virus is not well understood, but as mentioned in the section on CCHFV characteristics, glycoproteins Gn and Gc appear to be involved in recognizing receptor sites on cells susceptible to infection. It can infect various types of cells, particularly endothelial cells, immune system cells, and liver cells. Moreover, these glycoproteins seem to influence the infectivity of the virus, and there is a high probability that they are related to the high pathogenicity observed in humans [6].

CCHFV has pathological characteristics similar to other haemorrhagic viruses, such as Ebola or Dengue. Despite its relatively unknown pathology, it has been observed that the virus can weaken the host’s immune response, attacking and manipulating the cells responsible for activating it. Additionally, the virus’s replication causes deregulation of the host’s vascular system and lymphatic organs [1].

It appears that immune complexes are formed that activate the complement system, contributing to capillary damage and the beginning of renal and pulmonary failure seen in some cases of this disease. The infection also alters the endothelium, which becomes damaged, explaining the appearance of skin rashes. This deterioration could also affect normal blood coagulation, as it stimulates platelet aggregation, releases pro-inflammatory cytokines, and disrupts the coagulation cascade, causing coagulation inside blood vessels, resulting in thrombus and tissue damage, such as liver damage. In the advanced stages of the disease, immunological mechanisms such as cytokine storms are the main factor in pathogenesis [1,33,35].

Additionally, blood marker analysis shows an early elevation of creatine kinase along with high levels of the enzyme aspartate aminotransferase, indicating that the myalgia experienced by people with this infection may be directly related to muscle damage [33].

According to a study conducted in Turkey, patients with CCHF presented low platelet and white blood cell counts in their blood. As levels of liver enzymes (AST, ALT) and lactate dehydrogenase (LDH) were elevated, this may indicate liver damage [36].

## 6. Diagnosis of Crimean-Congo Hemorrhagic Fever Virus Infection

The WHO establishes the following tests as indicated for the diagnosis of this infection: virus isolation in cell cultures, enzyme-linked immunosorbent assay (ELISA), antigen detection, seroneutralization, and reverse transcription polymerase chain reaction (RT-PCR) [2].

Due to its high pathogenicity and the risk of contagion, the diagnosis of the CCHFV must be carried out in high-containment laboratories, generally BSL-4 [37].

Previously, methods such as intracerebral inoculation of a sample in mice were used to isolate the virus. Today, this test is no longer used because a rapid diagnosis is essential to properly address the disease, and this test was slow (between 5 and 10 days) [38].

- Cell Culture

It is possible to isolate the virus using cell lines such as LLC-MK2, Vero, BHK-21, SW13, and CER [1,34]. This is a less sensitive method than the one mentioned above, especially when dealing with low concentrations of viremia [39]. Additionally, it does not allow for rapid diagnosis, as the culture period can vary between 3 and 7 days, but it enables us to obtain high concentrations of the virus for additional studies.

- Antibody Detection

Enzyme-linked immunosorbent assay (ELISA) allow for the detection of antibodies in blood samples from patients. These assays are specific and more sensitive than immunofluorescence and seroneutralization tests, another class of serology-based diagnostic methods [1].

IgM antibodies appear between day 5 and 9 of the infection, during the acute phase of the disease (haemorrhagic phase). Nevertheless, IgG antibodies against CCHF appear later and are detected during the convalescent phase [37]. IgM antibodies are detectable for a period ranging from 3 weeks to 5 months after the symptoms end, while IgG antibodies are detectable up to 3 years later. This fact makes this assay very useful for determining the seroprevalence of the disease and the extent of exposure to the infection [34,37]. However, it is important to note that in individuals where the infection has been fatal, these tests may become negative results because antibody formation may not have occurred by the time of death [33].

- Molecular Methods

The method of choice for rapid diagnosis is RT-PCR because this test is effective during the first phase of the disease when symptoms are not yet very specific [37]. The test must be RT-PCR because CCHFV is an RNA virus, so its transcription to cDNA is essential [40]. One advantage of this test is that sample inactivation can be performed before the test, increasing safety and allowing the assay to be conducted at BSL-3 biosafety levels. A disadvantage is the wide genetic diversity characteristic of this virus, complicating the sensitivity of PCR. Segments of the S region of the virus genome are targeted as it is the most conserved [34].

A study conducted in Spain in 2023 proposes the use of an RT-LAMP test, an isothermal amplification technique that is very specific and sensitive. This test allows the detection of different genotypes of CCHFV, which is a significant advantage given the characteristic high genetic diversity. The sensitivity was compared, and the detection limit of RT-LAMP were found to be 103 times lower than that of RT-PCR. The study emphasizes that more research is needed to validate this assay [5].

The most suitable diagnostic test according to the phase of infection can be found in Figure 7.

## 7. Treatment and Prevention

### 7.1. Treatment

The mortality rate associated with this virus varies between 10% and 40%, with an average of approximately 30%. It is a severe disease as it can cause haemorrhagic fevers, thus highlighting the need for effective treatment.

The WHO recommends ribavirin as a treatment when there is a CCHFV infection [4], although there is controversy regarding the efficacy of this medication.

Another crucial part of the treatment is supportive therapy aimed at treating the patient’s symptoms and maintaining vital functions rather than fixing the cause of the disease. This includes the administration of platelets, fresh or frozen plasma, and red blood cell preparations [1].

Ribavirin is an antiviral drug that inhibits the replication of a wide range of viruses, both DNA and RNA, in vitro [41]. It is indicated for the treatment of CCHF and can be administered both orally and intravenously. The protocol indicates that treatment should begin with a high dose that is gradually decreased [3]. A recommended dosing regimen is an initial loading dose of 30 mg/kg, followed by 15 mg/kg every 6 h (4 × 1 g) for 4 days and then 7.5 mg/kg every 8 h (4 × 0.5 g) for 6 days [41].

It also appears that patients who receive ribavirin in the early stages of the disease are more likely to benefit from this medication, whereas in the later stages (haemorrhagic phase), when the virus’s pathogenicity is partly due to the body’s immune response, it may not be beneficial [35].

In a study conducted in Turkey, the mortality rate among study participants who received ribavirin was 2.8% compared to 4.5% in those who did not receive it. Additionally, no adverse effects were observed from its use. In the same study, it was suggested to treat all patients with severe disease and suspected CCHF infection with this medication, but it was noted that using it whenever there exists suspicion would not be cost-effective [36].

Another antiviral, Favipiravir, is being proposed as a possible treatment, but its efficacy is under study. In an in vivo trial conducted in mice, all animals that received this medication survived CCHFV infection [42].

Another measure that has been attempted as a treatment is the therapeutic use of antibodies derived from patients who have recovered from CCHF. This idea emerges because, in cases where patients do not survive, there is an absence of antibody formation. However, studies are needed to evaluate its efficacy [41].

### 7.2. Prevention

Given the limited treatment options available, prevention is essential to avoid exposure to the virus. The WHO recommends avoiding the risk of tick transmission by using protective clothing, acaricides, and repellents. If a tick is found, remove it safely and avoid areas with a high tick population, especially during seasons when they are more active. For livestock handlers and people working with animals, wearing gloves and protective clothing is recommended, especially when in contact with the animals’ blood or other fluids. To prevent person-to-person transmission, similar measures to those for other viruses should be taken: avoid physical and close contact if it is known that the person has CCHF and wash hands before and after visiting the sick [2].

It is crucial to emphasize safety measures in healthcare institutions when there is a patient with this disease due to the high risk of nosocomial infection [1]. In this regard, if prophylaxis is necessary due to high-risk contact, ribavirin is the drug used. The dose and duration are not detailed but are administered orally, and in the absence of specific guidelines, the same dose and duration previously mentioned for treatment are used [41].

Currently, there is no effective vaccine against CCHF. There is a chloroform-inactivated mouse brain-derived vaccine, but its safety and efficacy are not guaranteed [1].

## 8. Discussion

After describing all the relevant aspects of CCHFV due to the high pathogenicity of the virus, it is especially important to understand its current situation in Europe. This understanding ensures its prevention and permits an appropriate response to the potential spread of the agent in the future.

Regarding the current situation in Europe, as shown in the Section 4, only three countries (Bulgaria, Greece, and Spain) present autochthonous cases of CCHF. It is crucial to know the preventive measures and consider the presence of the disease to act appropriately.

On the one hand, focusing on countries completely free of CCHFV, there is a certain risk that rising temperatures due to climate change could result in a geographical expansion of the virus to these countries. This is due to the reservoir (ticks) reaching maturity earlier and achieving better survival rates [43].

On the other hand, countries such as Hungary, France, and Italy, where CCHFV has been found in ticks, should be particularly vigilant for the possible transmission of the virus to humans or animals (with presence already noted in animals in France). However, the risk of these countries becoming endemic depends on other factors such as climate and whether the environment is suitable for both the ticks and the animals that could become hosts [44].

In countries where the presence of the virus in humans has already been determined, the existence of the virus in the vector should be considered, especially during the months of high tick activity; those are spring and summer months from late April to August. It is very likely that the virus already resides as an endemic agent in both Bulgaria and Spain, leading to annual infection cases. Knowing this, it is crucial to have a good surveillance system in these countries that is capable of effectively and quickly detecting the infection to prevent and control the spread of this disease.

In Spain, the conditions exist for the continued appearance of autochthonous CCHF cases. The virus circulates in the country, and there are competent vectors and hosts that can amplify the cycle. Additionally, the climatic and environmental conditions are similar to those of other countries where the virus is endemic. The risk of cases occurring lies in areas where ticks are present [26]. Considering the numerous cases described in our country (Spain) and that one of them was detected thanks to a retrospective study, it would be very interesting to conduct a seroprevalence study in humans in areas where infected ticks are found. A study of this kind was previously conducted between 2013 and 2015, and no antibodies against CCHFV were detected [26]. However, now that the disease is more present, the results would likely be different, providing a better understanding of the true situation in the country.

Additionally, with the recent cases of CCHFV in Spain (2024), there have been five consecutive years of cases in the country. Knowing this data implies awareness that sporadic infection cases could be expected annually in the risk areas of the Iberian Peninsula. Most cases occur in June, July, and August (with a single case previously reported in Spain in April), and the latest case occurred in April. It is likely that the new climatological situation due to global warming could advance or extend the months of maximum tick activity.

This is a severe disease that can be fatal in some cases. However, it seems that with adequate containment measures, this disease can be managed without a significant impact on public health. For this virus to pose a significant public health risk, a large population of infected ticks and host animals must coexist in the same area. Additionally, for transmission to humans, there must be high contact with susceptible humans.

It is very important for physicians to consider the new epidemiological situation of the virus in their clinical suspicion in order to begin the treatment of choice and control measures as soon as possible to prevent further transmission. Moreover, as noted during the literature review, nosocomial infections are common, making it crucial to take preventive measures in case of infection.

While this review provides an updated overview of CCHF in Europe, several key questions merit further investigation to deepen our understanding of the disease dynamics. The relationship between CCHF in animal populations and human cases remains underexplored. Investigating how animal reservoirs correlate with human infections could offer crucial insights into transmission patterns and help identify potential hotspots for human outbreaks. Additionally, it is intriguing that CCHF has not spread more widely in regions where *Hyalomma marginatum* is present, despite suitable climatic conditions. This suggests there may be other limiting factors, such as ecological, behavioural, or epidemiological barriers, that need to be identified to understand why the disease remains relatively confined and to assess the conditions that could facilitate its expansion.

Furthermore, if CCHF has not fully utilized its current vector range, it raises important questions about its potential spread into projected vector ranges, especially under climate change scenarios. This underscores the need to examine environmental factors, host availability, and human interactions in relation to disease spread. The effectiveness of national surveillance systems and public health authorities is crucial in this context. Enhanced surveillance and improved diagnostic tools are essential for better tracking and managing CCHF cases. Additionally, fostering clinical suspicion among healthcare professionals will improve early detection and reporting. Finally, the concept of “empty niches”, where vectors are present but the disease is not, highlights the need to explore the specific ecological or epidemiological characteristics of these areas. Understanding how changes in land use, human behaviour, or vector control strategies might impact these dynamics could provide valuable insights. Addressing these questions, alongside strengthening surveillance and diagnostic capacities, will not only fill gaps in current knowledge but also inform more effective strategies for CCHF prevention and control in Europe and beyond.

## 9. Conclusions

Considering all the information gathered in this manuscript, it can be concluded that Crimean-Congo hemorrhagic fever virus is already present in Europe, being detected in animals in some countries and in humans in others, such as Spain. The virus exhibits high pathogenicity, and currently no effective treatment exists. This underscores the critical need for research to develop an effective treatment and a preventive vaccine. Epidemiological surveillance services in European countries must be proactive, as early diagnosis and robust prevention strategies can help avert future cases of infection.

## Figures and Tables

**Table 1:** Number of human cases of Crimean-Congo haemorrhagic fever in various European countries from 2017 to 2024 [9,16] *.

Country	2017	2018	2019	2020	2021	2022	2023	2024	TOTAL
Bulgaria	2	6	2	1	0	2	0	0	13
Greece	0	1	0	0	0	0	0	0	1
Spain	0	2	0	3	2	2	1	4	14

* The following countries have not reported human cases according to the ECDC for the period 2017–2024: Germany, Austria, Belgium, Cyprus, Croatia, Czech Republic, Denmark, Slovakia, Slovenia, Estonia, Finland, France, Hungary, Iceland, Ireland, Italy, Latvia, Liechtenstein, Lithuania, Luxembourg, Malta, Norway, Netherlands, Poland, Portugal, United Kingdom, Romania, and Sweden.

**Table 2:** Cases of Crimean-Congo haemorrhagic fever. Spain, 2015–2024 [27,28,29,30,31].

Case	Year ^1^	Month ^1^	Province	Transmission	Risk Factor	Age	Death
1	2013	May	Ávila	Tick	Rural walk	32	No
2	2016	August	Ávila	Tick	Rural walk	62	Yes
3	2016	August	Madrid	Nosocomial ^2^	Healthcare worker	50	No
4	2018	July	Badajoz	Tick	Hunting	74	Yes
5	2018	July	Salamanca	Tick	Farm animal	53	No
6	2020	June	Salamanca	Tick	Rural walk	69	No
7	2020	June	Salamanca	Tick	Animals	53	No
8	2020	August	Salamanca	Tick	Gardens	69	Yes
9	2021	April	Salamanca	Tick	Livestock farmer	59	No
10	2021	June	Leon	Tick	Rural walk	29	No
11	2022	June	Leon	Unknown	Environmental agent	51	Yes
12	2022	July	Leon	Tick	Hunting	49	No
13	2023	June	Salamanca	Tick	Unknown	66	No
14	2024	April	Salamanca	Tick	Rural walk	-	Yes
15	2024	July	Toledo	Tick	Rural walk	74	Yes
16	2024	July	Seville	Tick	Rural walk	46	No
17	2024	August	Caceres	Tick	-	65	No

^1^ Year and month according to symptom onset; ^2^ Nosocomial infection (person-to-person transmission).

## Data Availability

No new data were created or analyzed in this study. Data sharing is not applicable to this article.
